# Supplementary material for: In Vitro and In Situ Characterization of the Intestinal Absorption of Capilliposide B and Capilliposide C from Lysimachia capillipes Hemsl
Source: Molecules. 2019 Mar 28;24(7):1227. doi: 10.3390/molecules24071227 (PMC6479817; doi:10.3390/molecules24071227)
Supplement: Supplementary file 1 [file molecules-24-01227-s001.zip › Supplementary/Figure S legend.docx]

**Figure** **S1:** The standard curve of capilliposide B (CAPB) and capilliposide C (CAPC) across Caco-2 cell mono-layer was detected by liquid chromatography-tandem mass spectrometry （LC-MS）. There were four points over the concentration range of 1.0 - 250 ng/mL for CAPB (A) and CAPC (C) and seven points over the concentration range of 1.0-5000 ng/mL for CAPB (B) and CAPC (D).

**Figure S2:** Capilliposides (CAPs) inhibited the proliferation of caco-2 cells. Caco-2 cells were treated for 24 h with different concentrations (0, 10, 20, 40, 80, 120, 160 and 240 ug/mL) of CAPs. p < 0.05(*), comparison with the group of 0 ug/mL. All the results are expressed as mean ± S.D. (n=3).
