# Supplementary figures and images for: In Vitro and In Situ Characterization of the Intestinal Absorption of Capilliposide B and Capilliposide C from Lysimachia capillipes Hemsl
Source: Molecules. 2019 Mar 28;24(7):1227. doi: 10.3390/molecules24071227 (PMC6479817; doi:10.3390/molecules24071227)

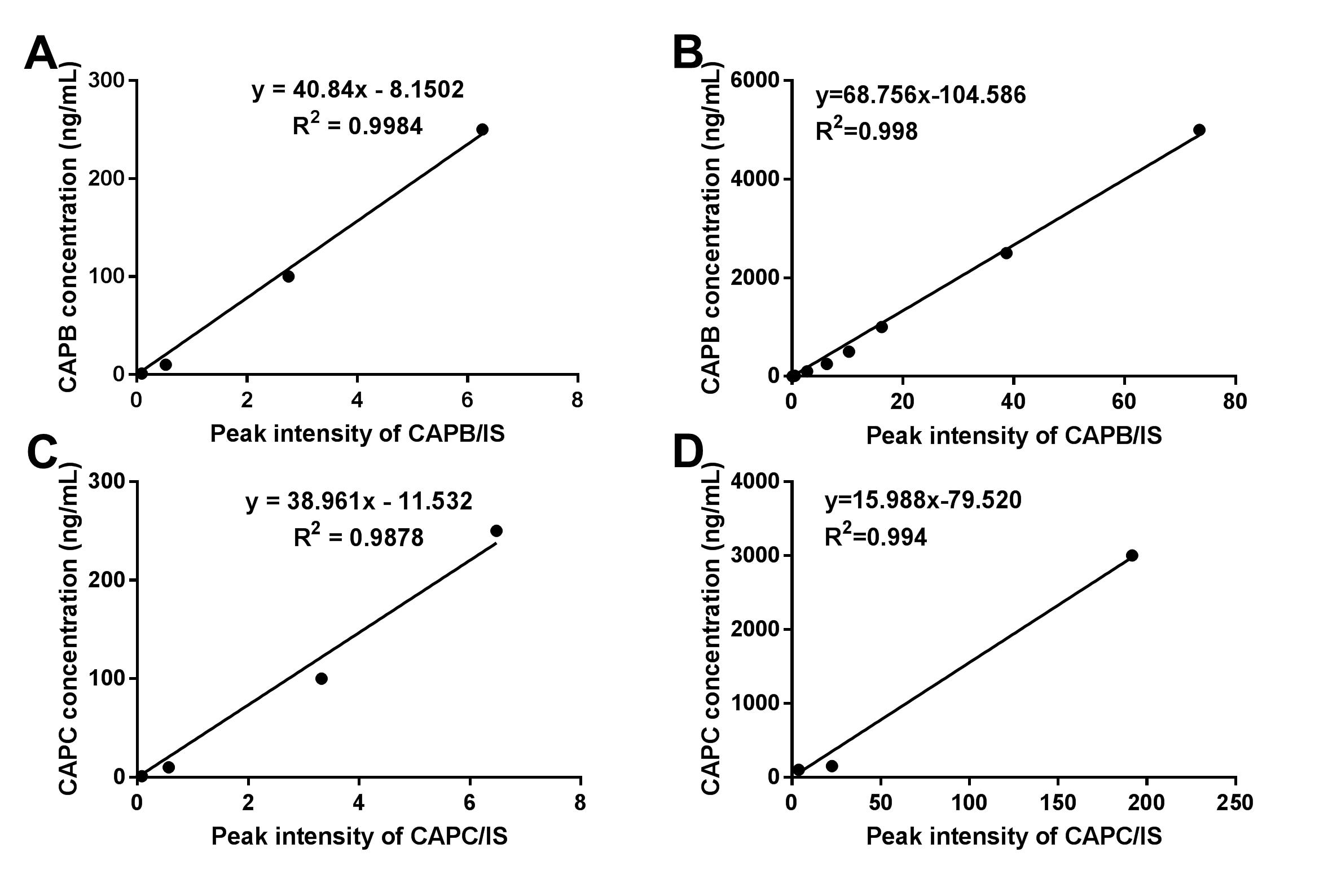

Supplement: Supplementary file 1 [file molecules-24-01227-s001.zip › Supplementary/Figure S1.jpg]

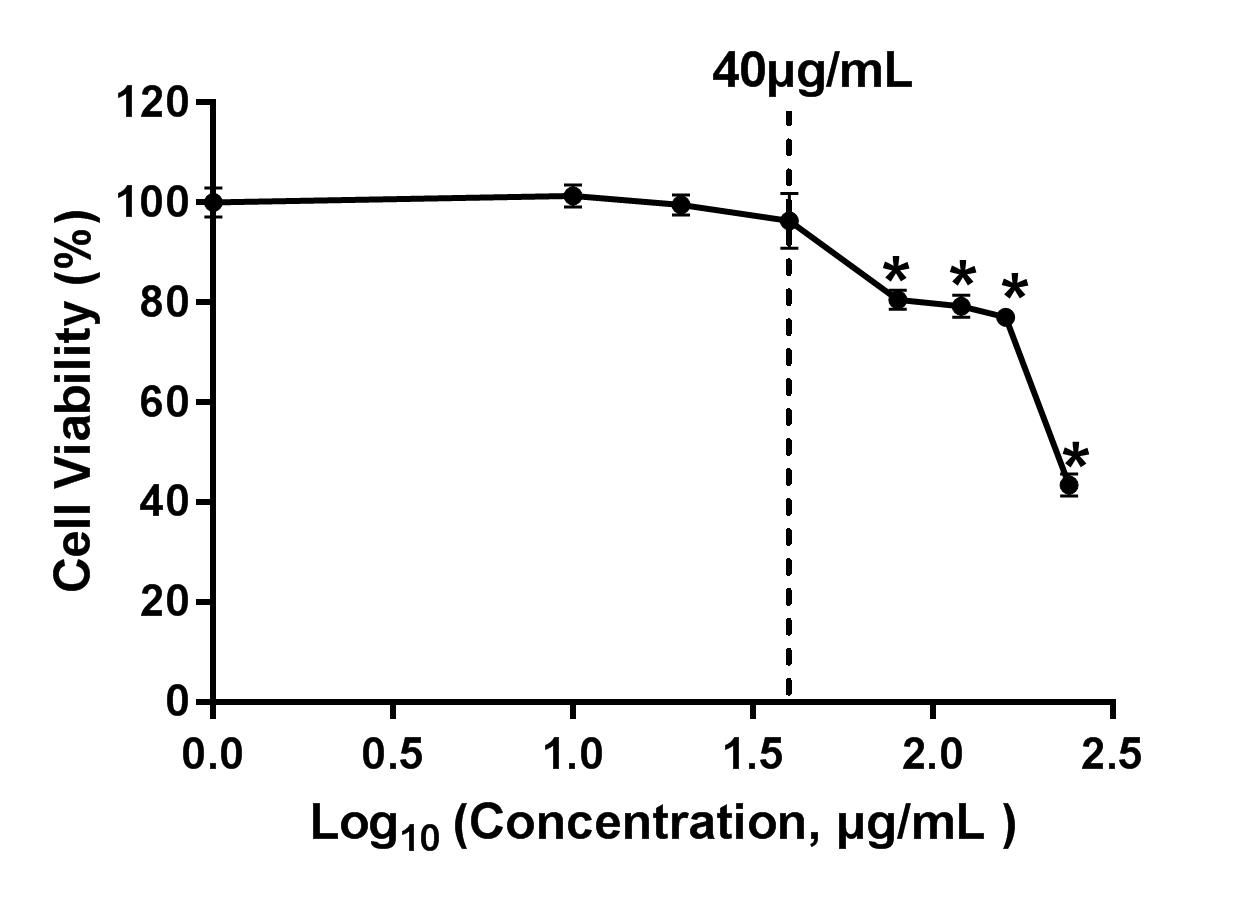

Supplement: Supplementary file 1 [file molecules-24-01227-s001.zip › Supplementary/Figure S2.jpg]
